# Efficacy, safety and pharmacokinetic of once-daily boosted saquinavir (1500/100 mg) together with 2 nucleos(t)ide reverse transcriptase inhibitors in real life: a multicentre prospective study

**DOI:** 10.1186/1742-6405-7-5

**Published:** 2010-03-17

**Authors:** Luis F López-Cortés, Pompeyo Viciana, Rosa Ruiz-Valderas, Juan Pasquau, Josefa Ruiz, Fernando Lozano, Dolores Merino, Antonio Vergara, Alberto Terrón, Luis González, Antonio Rivero, Agustin Muñoz-Sanz

**Affiliations:** 1 Servicio de Enfermedades Infecciosas, Hospitales Universitarios Virgen del Rocío, Instituto de Biomedicina de Sevilla, Seville, Spain; 2 Servicio de Medicina Interna, Hospital Universitario Virgen de las Nieves, Granada, Spain; 3 Sección de Enfermedades Infecciosas, Hospital Universitario Virgen de la Victoria, Malaga, Spain; 4 Sección de Enfermedades Infecciosas, Hospital Universitario de Valme, Seville, Spain; 5 Servicio de Medicina Interna, Hospital Juan Ramón Jimenez, Huelva, Spain; 6 Servicio de Medicina Interna, Hospital Universitario de Puerto Real, Puerto Real, Cádiz, Spain; 7 Servicio de Medicina Interna, Hospital de Jerez, Jerez de la Frontera, Cádiz, Spain; 8 Servicio de Medicina Interna, Hospital de Ceuta, Ceuta, Spain; 9 Sección de Enfermedades Infecciosas.Hospital Universitario Reina Sofía, Córdoba, Spain; 10 Sección de Enfermedades Infecciosas, Hospital Universitario Infanta Cristina, Badajoz, Spain

## Abstract

**Background:**

Ritonavir-boosted saquinavir (SQVr) is nowadays regarded as an alternative antiretroviral drug probably due to several drawbacks, such as its high pill burden, twice daily dosing and the requirement of 200 mg ritonavir when given at the current standard 1000/100 mg bid dosing. Several once-daily SQVr dosing schemes have been studied with the 200 mg SQV old formulations, trying to overcome some of these disadvantages. SQV 500 mg strength tablets became available at the end of 2005, thus facilitating a once-daily regimen with fewer pills, although there is very limited experience with this formulation yet.

**Methods:**

Prospective, multicentre study in which efficacy, safety and pharmacokinetics of a regimen of once-daily SQVr 1500/100 mg plus 2 NRTIs were evaluated under routine clinical care conditions in either antiretroviral-naïve patients or in those with no previous history of antiretroviral treatments and/or genotypic resistance tests suggesting SQV resistance. Plasma SQV trough levels were measured by HPLV-UV.

**Results:**

Five hundred and fourteen caucasian patients were included (47.2% coinfected with hepatitis C and/or B virus; 7.8% with cirrhosis). Efficacy at 52 weeks (plasma RNA-HIV <50 copies/ml) was 67.7% (CI_95_: 63.6 - 71.7%) by intention-to-treat, and 92.2% (CI_95_: 89.8 - 94.6%) by on-treatment analysis. The reasons for failure were: dropout or loss to follow-up (18.4%), virological failure (7.8%), adverse events (3.1%), and other reasons (4.6%). The high rate of dropout may be explained by an enrollement and follow-up under routine clinical care condition, and a population with a significant number of drug users. The median SQV Cmin (n = 49) was 295 ng/ml (range, 53-2172). The only variable associated with virological failure in the multivariate analysis was adherence (OR: 3.36; CI95, 1.51-7.46, p = 0.003).

**Conclusions:**

Our results suggests that SQVr (1500/100 mg) once-daily plus 2 NRTIs is an effective regimen, without severe clinical adverse events or hepatotoxicity, scarce lipid changes, and no interactions with methadone. All these factors and its once-daily administration suggest this regimen as an appropriate option in patients with no SQV resistance-associated mutations.

## Background

Saquinavir was the first protease inhibitor (PI) commercially available for the treatment of patients with HIV infection. Its oral bioavailability is markedly increased when concomitantly administered with low dose retainer, which allows for reduced dosing frequency and dosage. Ritonavir-boosted saquinavir (SQVr) at the standard dosing of 1000/100 mg twice daily has shown as effective as ritonavir-boosted-lopinavir, although requiring a higher pill burden when prescribed as the 200 mg hard or soft-gel capsules, which frequently leads to a bad compliance and high rates of therapy discontinuation [1,2]. In several guidelines for the treatment of HIV-1-infected patients, SQVr has remained as an alternative antiretroviral drug, probably due to its high daily pill burden, twice daily dosing and the requirement of 200 mg per day of ritonavir when given at the currently recommended dose [3,4]. On the other hand, several once-daily SQVr dosing schemes have been studied with these classic formulations, being 1600/100 mg/day the most frequently assessed regimen [5-8], but lower doses have also been tested, such as 1200/100 mg once-daily, with a favorable pharmacokinetic profile and clinical results [9-11].

SQV 500 mg strength tablets became available at the end of 2005. This formulation would facilitate a once-daily regimen (1500/100 mg) with fewer pills, although the experience with this dosage is still very scarce [12].

The aim of the present study was to assess the efficacy, safety and pharmacokinetics of once-daily SQVr 1500/100 mg plus 2 nucleos(t)ide reverse transcriptase inhibitors (NRTIs) in antiretroviral-naive patients or in those with no previous antiretroviral treatment history and/or genotypic resistance tests suggesting SQV resistance, under routine clinical care conditions.

## Results

### Baseline patients' characteristics

A total of 518 patients started a regimen of SQVr (1500/100 mg qd) plus 2 NRTIs at the mentioned centres during the mentioned period. One hundred and twenty patients (naïve, 14; experienced, 106) had a genotypic resistance test available just before starting SQVr. Four experienced patients had HIV protease mutations associated with SQV resistance (L90M) and were excluded from further analysis. Among the remaining cases, 33 (27.5%) had wild-type isolates, and 71 (59.1%) had resistance mutations in the reverse transcriptase (TAMs in 29 patients with a median (range) of 2 (1 -5); the K65R mutation was present in 5, the L74V in 6, and the M184I/V in 44; other mutations which confer resistance to non-nucleoside reverse transcriptase inhibitors was observed in 53 patients). Sixty eight patients had PI-related mutations, either minor mutations or polymorphism in most cases. One minor SQV-related mutation was present in 16 cases (L10I/V or I54V or I62V or A71T/V or V77I), and 3 minor resistance mutations (L10V, I62V and V77I) in 1 case. Genotypic resistance tests were not available in the rest of the patients, since amplification was not possible in cases with a VL <1000 copies/ml, or the test had not been requested in cases of treatment interruption for a long period, so that it was not expected to add relevant data.

The baseline characteristics of the 514 patients included in the analysis (group A: 50 naïve patients, group B: 80 patients who restarted ART after a temporary dropping out or lost to follow-up, group C: 81 with virological failure to a preceding PI- or NNRTI-based regimen, and group D or simplification group: 303) are summarized in table 1. Regarding the NRTIs used in combination with SQVr as part of the antiretroviral regimens, nearly 2/3 of the patients received tenofovir plus emtricitabine (TDF + FTC) or abacavir plus lamivudine (ABV + 3TC) (table 1).

**Table 1:** Patients' characteristics at inclusion (n = 514).

Groups	A n = 50	B n = 80	C n = 81	D n = 303
Age, years	39 (20 - 51)	41 (32 - 66)	41 (25 - 75)	41 (23 - 75)

Male	42 (84%)	62 (77.5%)	50 (61.7%)	223 (73.6%)

Weight, kg	68 (50 - 102)	64 (42 - 98.5)	65.5 (36 - 111)	65.9 (39 - 121)

Risk factor for HIV				
IV drug use	23 (46%)	64 (80%)	53 (65.4%)	185 (61.1%)
Hetero/homosexual	27 (64%)	15 (20%)	26 (32.1%)	110 (36.3%)
Blood products transfusion			1 (1.2%)	3 (1%)
Unknown			1 (1.2%)	5 (1.7%)

Methadone treatment	11 (22%)	36 (45%)	20 (24.7%)	56 (18.5%)

Active illegal drug use	4 (8%)	14 (17.5%)	8 (9.9%)	17 (5.6%)

CD4/μl	140 (4 - 563)	227 (4 - 546)	277 (14 - 923)	475 (27 - 1196)

HIV-RNA log_10 _cop./ml	5.16 (2.0-6.36)	4.61 (2.05-6.54)	3.52 (2.04-4.64)	< 1.69 (< 1.69-2.45)

Clinical category C	10 (20%)	26 (32.6%)	28 (34.6%)	98 (32.4%)

Nadir CD4/μl	152 (4 - 417)	120 (1 - 476)	120 (1 - 815)	130 (1 - 825)

Previous ART (months)	-	46 (1 - 164)	38 (1 -192)	71 (1 - 269)

Associated NRTIs				
TDF + FTC	34 (68%)	46 (57.5%)	28 (34.6%)	110 (36.3%)
ABV + 3TC	8 (16%)	15 (18.8%)	14 (17.3%)	78 (25.7%)
ddI + 3TC	5 (10%)	3 (3.8%)	5 (6.2%)	37 (12.2%)
Others	3 (6%)	16 (20.8%)	34 (41.9%)	78 (25.7%)

Chronic viral hepatitis	22 (44%)	63 (78.8%)	48 (59.3%)	190 (62.7%)
HCV	21 (42%)	55 (68.8%)	45 (55.5%)	185 (61.0%)
HBV	1 (2%)	2 (2.5%)	2 (2.5%)	2 (0.7%)
HCV + HVB	-	2 (2.5%)	1 (1.2%)	3 (1.0%)

Cirrhosis	4 (8%)	4 (5%)	6 (7.4%)	26 (8.6%)

ART: antiretroviral treatment. PIs: protease inhibitors. NNRTIs: non-nucleos(t)ide reverse transcriptase inhibitors. M: median. Group A: naive patients. Group B: patients who restarted antiretroviral therapy after dropping out. Group C: patients with virological failure to a PI- or NNRTI-based regimen. Group D: patients who simplified a PI-based regimen to an once daily regimen or had toxicity to a previous regimen based on PIs or NNRTIs. Variables expressed as no. (%) or median (range).

### Virological and immunological response

**Figure 1:**
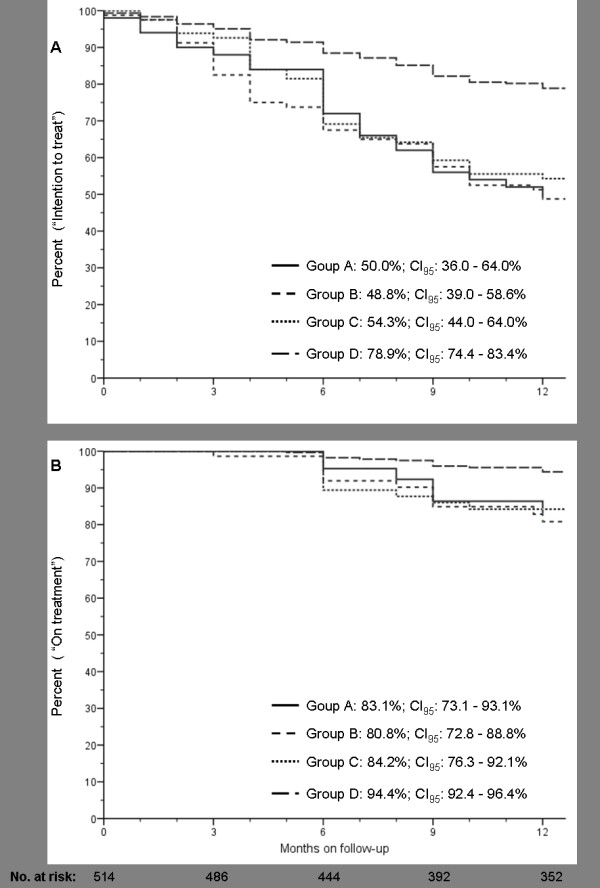
**A) Kaplan-Meier estimates of the percentage of patients without treatment failure (intention-to-treat) and B) without virological failure (on treatment) through week 52. **Groups A: antiretroviral-naïve patients, B: patients who restarted ART after a temporary dropping out or lost to follow-up, C: patients with virological failure to a preceding PI- or NNRTI-based regimen, and D: those with an undetectable viral load who simplified a PI-based regimen or had toxicity to a previous regimen based on PIs or NNRTIs.

For the whole of patients, the treatment efficacy at 52 weeks was 67.7% (CI_95_: 63.6 - 71.7%) by ITT, and 92.2% (CI_95_: 89.8 - 94.6%) by on-treatment analysis (figure 1). In both cases, the efficacy was higher in the simplification group (*p *= 0.000, and 0.01, respectively) and with no significant differences between the other groups. By ITT, 135 patients (26.2%) failed because of treatment dropout or loss to follow-up in 95 cases (18.4%), AEs in 16 cases (3.1%), and other reasons (imprisonment, move, drug interactions and death) in 24 patients (4.6%). Virological failure occurred in 40 patients (7.8%): group A, 6/50 (12%), group B, 10/80 (12.5%), group C, 10/81 (12.3%), and group D, 14/303 (4.6%). In 17 of them VL had not achieved <50 copies/ml after 24 weeks of treatment (group A, 4; group B, 7, group C, 7), and the other 23 showed a confirmed viral load >200 copies/ml after a previously undetectable viral load. The variables associated with virological failure in the univariate analysis were baseline VL >100000 copies/ml, baseline CD4 count <200/μl, and non-adherence. No relationship was found between virological outcome and either treatment group, baseline PI-related mutations, earlier PI failure, methadone treatment or active illegal drug use. The only variable associated with virological failure in the multivariate analysis was adherence (OR: 3.36; CI_95_, 1.51 - 7.46, p = 0.003).

Genotypic resistance testing was available in 18 patients at the moment of virological failure, but only in 11 of them VL was high enough to allow amplification. In 2 of them a wild type virus was observed; RT and protease genotypes at failure are reported in table 2. The median increase from baseline in CD4 cell counts at week 52 was 114 cells/μl; this increase was inversely proportional to baseline CD4 counts. Thus, it was 224 cells/μL (range, -108 to 542) in group A, 130 cells/μL (range, -45 to 812) in group B, 88 (range, -290 to 565) in group C, and 58 cells/μL (range, -389 to 571) in the "simplification" or group C.

**Table 2:** Genotypic resistance tests at failure according to treatment groups.

Patient	Group	NRTIs	Retrotranscriptase	Protease
1	Naïve patients	ABV + 3TC		10I, L63P

2		TDF + FTC	M184V	L63P

3		TDF + FTC	M184V	L63P, M46I, F53L

4	Restarting ART	ABV + 3TC		10I, 63P

5		TDF + ddI	M184V	D30N

6		AZT + ddI	K103N	V77I

7		TDF + FTC	K70R, T215F, 219Q	L63P

8		ZDV + TDF	M41L, D67N, K70R, L210W, T215Y	E35D, M36I, F53L, D60E, L63P, A71V, I84V

9	Previous failure	ZDV + TDF	L215Y	

In 2 additional patients a wild type virus was observed. NRTIs: nucleos(t)ide reverse transcriptase inhibitors administered together with ritonavir-boosted saquinavir (1500/100 mg once daily). ABV: abacavir, ddI: didanosine, 3TC: lamivudine, FTC: emtricitabine, TDF: tenofovir, ZDV:zidovudine.

### Adverse events

**Figure 2:**
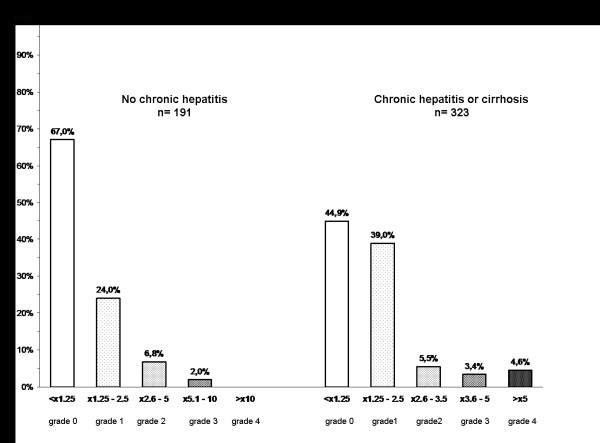
**Proportion of patients (n) who developed aminotransferase elevations in any determination throughout the follow-up**.

The most frequently reported AEs were grade 1-2 digestive symptoms (47 cases; 9.1%). Rash appeared in 4 patients (0.7%), in 3 of them it was related with abacavir. Grade 1 serum creatinine elevations occurred in 5 patients, 4 of them concomitantly receiving tenofovir. Five of the 6 cases of lypodystrophy were patients from the "simplification" group and 1 from the "ART-restart" group (table 3). These AEs caused treatment withdrawal in 15 patients (3%): digestive symptoms (n = 13), lypodystrophy (n = 1), and serum creatinine increase (n = 1). Figure 2 shows the proportion of patients with increased aminotransferases levels in any determination throughout the follow-up, although none of those cases was symptomatic and the alterations observed were transient and improved without treatment discontinuation in every case.

**Table 3:** Adverse events during the follow-up.

Clinical adverse events	[no. (%)]
Nausea or vomiting and/or abdominal discomfort	38 (7.4)
Diarrhea	9 (1.7)
Lypodystrophy	6 (1.1)
Rash	4 (0.8)
Fatigue	6 (1.1)
Depression	4 (0.8)
Dizziness	4 (0.8)
Headache	1 (0.02)
Hepatic encephalopathy	1 (0.02)
Insomnia	1 (0.02)
Seizure	1 (0.02)
**Laboratory adverse events**	
	
AST or ALT increase (grade 2-4)	62 (12.0)
SCr elevation (grade 1)	5 (1.2)
Anemia and thrombocytopenia	1 (0.02)

SCr: serum creatinine.

In 354 patients who had a complete lipid profile throughout the 52 weeks of follow-up, the median change at week 52 in the total cholesterol value from baseline was 1 mg/dl (IQR, -21 to 22); in LDL-cholesterol, -1 mg/dl (IQR, -20 to 17), in HDL-cholesterol, 0 mg/dl (IQR, -8 to 7), and in triglyceride levels -9 mg/dl (IQR, -41 to 27), respectively. Among the patients starting or restarting ART (groups A and B), the median change at week 52 in total cholesterol value from baseline was 12 mg/dl (IQR, -7 to 32), in LDL-cholesterol 2 mg/dl (IQR, -19 to 23), in HDL-cholesterol 8 mg/dl (IQR, -1 to 16), and in triglyceride levels -6 mg/dl (IQR, -42 to 39), respectively (table 4). No symptoms of methadone withdrawal were observed.

**Table 4:** Lipid levels throughout the follow-up.

Lipid levels	Baseline	Month 3	Month 6	Month 9	Month 12
**All patients**	(n = 514)	(n = 433)	(n = 388)	(n = 352)	(n = 355)

Total cholesterol, mg/dl	169 (23-427)	168 (31-298)	168 (36-340)	171 (61-348)	173 (54-297)
Individuals with ≥ 240 mg/dl	38 (7.3%)	21 (4.8%)	25 (6.4%)	25 (7.1%)	22 (6.2%)

LDL cholesterol, mg/dl	96 (20-320)	95 (20-181)	95 (15-210)	95 (20-215)	96 (20-189)
Individuals with ≥ 160 mg/dl	25 (4.8%)	14 (3.2%)	15 (3.8%)	16 (4.5%)	12 (3.4%)

Total triglycerides, mg/dl	133 (22-1637)	134 (13-976)	129 (37-881)	139 (39-1708)	128 (36-1664)
Individuals with ≥ 200 mg/dl	103 (20.0%)	108 (24.9%)	83 (20.6%)	84 (23.8%)	67 (18.8%)
Individuals with ≥ 400 mg/dl	17 (3.3%)	22 (5.0%)	11 (2.8%)	12 (3.4%)	13 (3.6%)

**Naive and ART-restarting patients (groups A and B)**	(n = 130)	(n = 102)	(n = 87)	(n = 79)	(n = 69)

Total cholesterol, mg/dl	148 (53-285)	161 (31-298)	162 (61-340)	167 (61-256)	164 (69-266)
Individuals with ≥ 240 mg/dl	2 (1.5%)	2 (1.9%)	3 (3.4%)	1 (1.2%)	4 (5.8%)

LDL cholesterol, mg/dl	84 (20-211)	91 (20-178)	91 (15-210)	94 (11-156)	90 (20-157)
Individuals with ≥ 160 mg/dl	2 (1.5%)	3 (3.4%)	3 (3.4%)	0 (0%)	0 (0%)

Total triglycerides, mg/dl	115 (22-1637)	118 (44-882)	125 (37-680)	113 (41-484)	120 (36-378)
Individuals with ≥ 200 mg/dl	26 (5.0%)	16 (3.7%)	14 (3.6%)	13 (3.7%)	5 (1.4%)
Individuals with ≥ 400 mg/dl	3 (0.5%)	4 (0.9%)	2 (0.5%)	2 (0.05%)	1 (0.02%)

Variables expressed as no. (%) or median (range).

### Plasma Saquinavir levels

SQV trough levels were available in 49 patients (41 M, 8 F) weighing a median of 65.5 kg (range, 44 - 98.5). Twenty four (49%) of them were affected by viral chronic hepatitis and 9 (18.3%) by cirrhosis. The median SQV trough level was 295 ng/ml (range, 53 - 2172); four patients (8.1%) had values under 100 ng/ml, and 3 of them had a satisfactory virological response. No correlations were found between SQV levels and either weight, gender or the presence of chronic hepatitis and/or cirrhosis.

## Discussion

Although the approved dosage of SQVr is 1000/100 mg twice daily, several once-daily schemes (1200/100, 1600/100 and 2000/100 mg/day) plus 2 NRTIs have demonstrated a good virological efficacy in patients with no SQV resistance mutations [5-11]. From a pharmacokinetic point of view, once-daily SQVr 1500/100 mg yielded SQV trough levels similar to those observed with a dose of 1600/100 mg daily [14-20], exceeding both the IC_95 _value (25 ng/ml) for wild HIV-1 isolates and the estimated trough level required to obtain the half-maximal antiviral response (EC_50_: 50 ng/ml) [21]. In our study, SQV trough levels in plasma were similar between patients with and without chronic viral hepatitis or cirrhosis, as previously reported in the absence of liver function impairment [22]. Four out of 49 sampled patients had a SQV C_*min *_below 100 ng/ml, and 3 of them had a satisfactory virological response. Although 100 ng/ml is suggested as the minimum target trough concentration for wild-type HIV-1 [4], this value has not been corroborated yet in clinical trials, especially in the presence of other drugs with activity against HIV. Moreover, SQV has been demonstrated to accumulate in peripheral mononuclear blood cells *in vivo*, resulting in a median intracellular drug accumulation ratio of 2.75-3 as compared with that in plasma, suggesting that intracellular exposure to SQV may be a better predictor of the virological response to therapy [8]. Thus, our pharmacokinetic data support the 1500/100 mg qd dose as adequate for patients without SQV resistance mutations.

The efficacy in the ITT analysis in our series (65.7%) is similar to that observed in the MaxCmin2 trial and in the Gemini study with a 1000/100 bid SQVr dosing [1,2], although with a higher failure rate due to dropout, loss to follow-up or other causes not related with the antiretroviral regimen itself, but mainly explained by the fact that patients were enrolled and followed up under routine clinical care conditions, without the selection criteria used in clinical trials, and with a significant number of drug users, a population known to be particularly non-adherent. Actually, many of them had previously discontinued other antiretroviral treatments. The virological failure rate observed was just 8.5%, being adherence the only variable related in the multivariate analysis. In the OT analysis, the efficacy raised to 90.4%, with similar results regardless of baseline VL and CD4 counts. The presence of more adherent patients in the "simplification" group may be the cause of the higher efficacy observed in these patients. Although the efficacy of a once daily dosing of SQV/r has mainly been shown to be effective in an Asian population whom in general have lower body weight, our results show convincing data that in Caucasians with higher body weight, the once daily dosing has also good efficacy.

Evaluation of the available genotypic tests from patients with virological failure revealed a low incidence of selection of protease inhibitors major/minor resistance mutations following treatment with SQV/r, which is consistent with previous observations made for boosted protease inhibitors, mainly in naive patients (3).

The combination of once-daily SQVr was well tolerated during the 52-week follow-up, with no clinical grade 3 or 4 adverse events recorded. Only 12 patients (3.0%) changed their regimen because of AEs. Although lypodystrophy occurred in 6 patients after switching treatment to SQVr, this may reflect long-term antiretroviral drug exposure rather than an effect caused only by this regimen, since 5 out of 6 patients belonged to the "simplification"group, and the remaining one to the ART-restart group. Particularly meaningful are the results regarding liver toxicity in a population with 58.7% of the patients presenting chronic hepatitis (VHC: 95,5%), 10,7% of them being cirrhotic. Among patients with and without chronic hepatitis and/or cirrhosis, only 1 (0.6%) and 13 cases (5.4%), respectively, developed grade 3-4 transaminase increases. Moreover, none of these cases was symptomatic, and the alterations observed were transient and improved without treatment discontinuation in every case, which may indicate that much of these elevations could be due to the natural evolution of chronic hepatitis and/or cirrhosis rather than caused by the treatment. Also, it is remarkable that patients receiving this regimen showed no changes, or just negligible increases, in the levels of total cholesterol, LDL cholesterol, and triglycerides.

The absence of relevant pharmacokinetic interactions between SQVr and methadone is an additional advantage in patients on methadone maintenance therapy [23,24].

We are aware that the open-label characteristics of the study, the heterogeneity of the analyzed population and the lack of available genotypic resistance tests in some of the patients at baseline and after virological failure are limitations of our study, although they reflect the real-life clinical setting.

## Conclusions

This open-label multicentre study suggests that SQVr (1500/100 mg) once-daily plus 2 NRTIs is an effective regimen, without severe clinical adverse events or hepatotoxicity, scarce lipid changes, and no interactions with methadone. All these factors and its once-daily administration make this regimen make this regimen worth to be considered as an alternative in patients with no SQV resistance-associated mutations. In addition, the 1500/100 mg qd dosage is one of the cheapest PI combinations and, given that the patent will soon expire, a more affordable generic formulation would then be available, making it possible a more extended use of this drug.

## Methods

### Study Population and design

From November 2005 to May 2007, HIV-1 infected patients older than 18 years attended at the HIV clinics in 17 hospitals from Andalusia, Ceuta and Extremadura (Spain), and scheduled to receive a regimen of SQVr 1500/100 mg once-daily plus 2 NRTIs, were consecutively enrolled in this observational, prospective, single-arm, open-label study. NRTIs prescribed as part of HAART were selected by the responsible physicians on the basis of previous antiretroviral treatments (ART) and/or genotypic resistance testing. Patients were enrolled and followed-up under routine clinical care conditions, and no entry restrictions were made except for pregnancy, history of previous ART and/or genotypic resistance tests suggesting resistance to SQV according to the 2005 International AIDS Society [13], and the concomitant use of drugs with potential adverse interactions with SQV pharmacokinetics, such as rifampin. Patients who received pegylated interferon-alpha plus ribavirin for chronic hepatitis C during their follow-up were excluded hereinafter from CD4 cell counts changes analysis since this treatment usually modifies the hematologic profiles and CD4 cell counts. Patients were initially classified according to previous ART in the following groups: A) antiretroviral-naïve patients, B) patients who restarted ART after a temporary dropping out or lost to follow-up, C) patients with virological failure to a preceding protease inhibitor (PI)- or non-nucleoside reverse transcriptase inhibitors (NNRTI)-based regimen, and D) those with an undetectable viral load who simplified a PI-based regimen to an once-daily regimen or had toxicity to a previous regimen based on PIs or NNRTIs. The study was approved by the Regional Ethics Committee for Clinical Research of the Community of Andalusia, and conducted according to the principles contained in the Declaration of Helsinki. All patients gave an informed consent. The patients' inclusion was censored in May 2007 to allow a minimum of 12 months of follow-up.

### Follow-up, assessments and endpoints

Patients' assessment was performed at baseline, after the first month on treatment and every three months thereafter, including adverse effects (AEs), biochemical and hematologic profiles, flow cytometric count of CD4/μl and plasma HIV-1-RNA (VL) measured by polymerase chain reaction (lower detection limit: 50 copies/ml. Amplicor HIV-1 Monitor test version 1.0; Roche Diagnostic Systems). Adherence was evaluated by personal interview at each following visit. Efficacy at 52 weeks, analyzed by intention-to-treat (ITT), was the primary clinical endpoint. Virological failure was defined as inability to suppress plasma VL to <50 copies/ml after 24 weeks on treatment, or a confirmed viral load >200 copies/ml in patients who had previously achieved a viral suppression or had an undetectable viral load at inclusion. If confirmed, the time of the first measurement meeting the failure criteria was selected as the time of failure. Secondary outcomes included virological efficacy according to on-treatment (OT) analysis, changes in CD4 cell counts, incidence of AEs and lipid profiles. The changes in serum ALT and AST from pre-treatment levels to the highest level during treatment were categorized via a standardized toxicity grade scale, modified from that used by the AIDS Clinical Trials Group. Patients with pre-treatment serum AST and ALT levels within normal range (AST <35 IU/L and ALT <31 IU/L) were classified according to the changes observed with respect to the upper limit of normal (ULN): grade 0 (<1.25 ULN); grade 1 (1.25-2.5 × ULN); grade 2 (2.6-5 × ULN); grade 3 (5.1-10 × ULN); and grade 4 (>10 × ULN). In patients with chronic viral hepatitis or cirrhosis, toxicity was classified according to changes relative to baseline values rather than ULN: grade 0 (<1.25 × baseline); grade 1 (1.25-2.5 × baseline); grade 2 (2.6-3.5 × baseline); grade 3 (3.6-5 × baseline); and grade 4 (>5 × baseline). Genotypic resistance tests were performed in patients with virological failure whenever viral load levels allowed. Patients missing two consecutive scheduled visits were considered lost to follow-up.

### Blood sampling and determination of saquinavir concentrations

Blood samples for SQV plasma levels were obtained 24 ± 0.3 hours post-dose, after at least one month on treatment, from random patients who usually took SQVr in the morning and who were included in the study at Hospitales Universitarios Virgen del Rocío. Plasma samples were stored frozen at -80°C for determination of SQV by high-performance liquid chromatographic assay according to a validated method [10].

### Statistical analysis

Descriptive statistic was used for demographic, epidemiological and clinical data, prior ARTs, CD4 cell count, viral load and SQV trough concentrations. Kaplan-Meier plots were produced for the 'time to event' analyses and comparisons among the 3 treatment groups were made using the log-rank test. The relationships between virological failure and different variables were assessed by the chi-square test for qualitative variables and by the Spearman's rank-correlation coefficients for quantitative variables. The variables tested by univariate analysis as predictors of virological failure with a p value <0.1 were included in a multivariate analysis to identify possible independent predictors of virological failure. Statistical calculations were performed with the Statistical Product and Service Solutions for Windows (15.0 version, SPSS, Chicago, IL).

## Competing interests

LFLC, PV, FZ, and AR have received unrestricted funds for research and honoraria for speaking at symposia organized on behalf of Abbott laboratories (Spain), Bristol-Myers Squibb, GlaxoSmithkline, Gilead Sciences, Janssen-Cilag España, Merck Sharp & Dohme España, and Roche Pharma SA. Other authors: none to declare.

## Authors' contributions

Conception, design, analysis, interpretation of the data, drafting of the article and obtaining of funding: LFLC.

Provision of study materials or patients: LFLC, PV, JP, JR, FL, DM, AV, AT, LG, AR, AMS,

Determination of saquinavir plasma concentrations: RRV.

Critical revision of the article for important intellectual content: PV, RRV, JP, JR, FLo, DM, AV, AT, LG, AR, and AMS.

Final approval of the article: LFLC, PV, RRV, JP, JR, FL, DM, AV, AT, LG, AR, and AMS

Collection and assembly of data: LFLC, PV, JP, JR, FL, DM, AV, AT, LG, AR, AMS.

All authors have read and approved the final manuscript.
